# Aberrant Bone Homeostasis in AML Is Associated with Activated Oncogenic FLT3-Dependent Cytokine Networks

**DOI:** 10.3390/cells9112443

**Published:** 2020-11-09

**Authors:** Isabel Bär, Volker Ast, Daria Meyer, Rainer König, Martina Rauner, Lorenz C. Hofbauer, Jörg P. Müller

**Affiliations:** 1 Institute of Molecular Cell Biology, Center for Molecular Biomedicine (CMB), Jena University Hospital, 07745 Jena, Germany; isabel.baer@uni-jena.de; 2 Institute for Clinical Chemistry, Medical Faculty Mannheim, Heidelberg University, 69117 Heidelberg, Germany; Volker.Ast@medma.uni-heidelberg.de; 3 Center for Infectious Diseases and Infection Control, Jena University Hospital, 07745 Jena, Germany; daria-meyer@web.de (D.M.); rainer.koenig@uni-jena.de (R.K.); 4 Integrated Research and Treatment Center, Center for Sepsis Control and Care (CSCC), 07745 Jena, Germany; 5 Department of Medicine III & Center for Healthy Aging, Technical University Dresden, 01069 Dresden, Germany; Martina.Rauner@uniklinikum-dresden.de

**Keywords:** acute myeloid leukaemia (AML), FMS-like tyrosine kinase 3 (FLT3), haematopoietic niche, haematopoiesis, bone remodelling, osteoclast (OC), osteoblast (OB), osteoblastogenesis

## Abstract

Acute myeloid leukaemia (AML) is a haematopoietic malignancy caused by a combination of genetic and epigenetic lesions. Activation of the oncoprotein FLT3 ITD (Fms-like tyrosine kinase with internal tandem duplications) represents a key driver mutation in 25–30% of AML patients. FLT3 is a class III receptor tyrosine kinase, which plays a role in cell survival, proliferation, and differentiation of haematopoietic progenitors of lymphoid and myeloid lineages. Mutant FLT3 ITD results in an altered signalling quality, which causes cell transformation. Recent evidence indicates an effect of FLT3 ITD on bone homeostasis in addition to haematological aberrations. Using gene expression data repositories of FLT3 ITD-positive AML patients, we identified activated cytokine networks that affect the formation of the haematopoietic niche by controlling osteoclastogenesis and osteoblast functions. In addition, aberrant oncogenic FLT3 signalling of osteogenesis-specific cytokines affects survival of AML patients and may be used for prognosis. Thus, these data highlight the intimate crosstalk between leukaemic and osteogenic cells within the osteohaematopoietic niche.

## 1. Introduction

Haematopoietic stem and progenitor cells (HSPCs) reside in a specialized microenvironment, which is essential to support their function and to maintain a balance between self-renewal and differentiation. The formation of this niche in the bone marrow (BM) is tightly associated with bone remodelling and endochondral ossification [1]. Cells in osteoclast (OC) and osteoblast (OB) lineages define the scaffold for the HSPC niche by communicating in a bidirectional fashion through cell–cell contact, diffusible paracrine factors, and cell–bone matrix interactions. The BM niche provides the scaffold for cell populations that are directly involved in haematopoiesis and also represents the home of leukaemic blasts [2]. Thus, this microenvironment contributes to progression of haematological malignancies [3]. It has been demonstrated that an altered BM microenvironment can even initiate leukaemogenesis in healthy cells [4]. Osteoporosis with fragility fractures are frequently encountered in adults with haematological diseases after conventional therapy [5,6,7]. After HSPC transplantation, the prevalence of osteoporosis increases dramatically due to frequent bone loss [8]. 

OBs, which are required for the formation of the HSPC niche by synthesising bone matrix, derive from mesenchymal skeletal stem cells [9,10]. OCs, which have a unique role in bone resorption and directly contribute to haematopoietic progenitor mobilization, originate from erythro-myeloid progenitors [11]. OC formation requires the presence of RANKL (receptor activator of nuclear factor-κB ligand, encoded by *TNFSF11*) and M-CSF (macrophage colony-stimulating factor, encoded by *CSF1*). OC differentiation is inhibited by osteoprotegerin (OPG, encoded by *TNFRSF11B*), which is produced by OB and other cell types like endothelial cells and binds to RANKL [12], thereby preventing interaction with RANK (encoded by *TNFRSF11A*), the RANKL-binding receptor abundantly expressed by OCs [13]. Thus, the equilibrium between RANKL and OPG regulates the OC-mediated bone degradation [14] and is therefore an important system that determines bone mass and structure [15,16]. 

Additionally to M-CSF and RANKL, FLT3 ligand FL (encoded by *FLT3LG*) has been shown to drive osteoclastogenesis [17]. Work focusing on factors controlling the development of rheumatoid arthritis revealed that bone loss of rheumatoid arthritis patients was associated with elevated FL levels. Moreover, FL knockout mice failed to develop arthritis in a collagen-induced arthritis model [18,19,20]. Local knee exposure to FL aggravated arthritis in mice [18]. Thus, the above findings suggest that the FL–FLT3 signalling axis has proosteoclastogenic properties [21]. The receptor tyrosine kinase FLT3 plays a role in proliferation and differentiation of B-cell progenitors, myelomonocytic and dendritic cells, as well as in the maintenance of pluripotent haematopoietic stem cells [22]. Activating mutations of FLT3 causally contribute to leukaemogenesis [23]. Internal tandem duplications (ITDs) in the juxtamembrane domain of the receptor induce ligand-independent constitutive signalling and are associated with high relapse rates and poor overall survival of AML patients. A recent mouse study revealed that oncogenic FLT3 ITD leads to alterations in bone morphology. Inactivation of antagonistic receptor protein tyrosine phosphatase CD45 consequently increased oncogenic FLT3 ITD activity, which resulted in an exacerbated bone phenotype. Here, features of ectopic bone formation in spleen and kidney were observed. This phenotype suggests a previously unappreciated capacity of FLT3-ITD (and presumably FLT3) to regulate bone development/remodelling, which is under negative control of CD45/Ptprc [24].

Taken together, OB and OC commitment and differentiation are controlled by a close interwoven complex of components. Aberrant bone structures and ectopic bone formation of FLT3 ITD-positive mice demonstrated that signalling networks controlling haematopoiesis also have an impact on bone homeostasis [24]. By comparing gene expression data of FLT3 ITD-positive AML patient samples to FLT3 wild type (WT) AML patients, we addressed the question of how FLT3 ITD affects the expression of genes controlling osteoclastogenesis and the formation and activity of OBs. In addition, factors that are crucial for the prognosis of AML patients were identified. These data will help to identify prognostic factors that contribute to the development of the haematological disease. A detailed understanding of how FLT3/FLT3 ITD is involved in the crosstalk of leukaemic blast cells with the BM microenvironment may provide key insights into bone disease in AML and could open ways for prevention and therapy [25].

## 2. Material and Methods

### 2.1. Gene Expression Datasets

Transcriptomic datasets derived from blasts and mononuclear cell from bone marrow or peripheral blood were extracted from the Gene Expression Omnibus (GEO) repository at the National Center for Biotechnology Information (NCBI) (http://www.ncbi.nlm.nih.gov/ge, accessed on 1 March 2018). We used the datasets of Metzeler [26] (GEO ID GSE12417), Verhaak [27] (GEO ID GSE6891), and Valk [28] (GEO ID GSE1159). The Metzeler dataset consists of two independent cohorts (GEO ID GSE12417-GPL570 and GSE12417-GPL96) comprising 79 and 163 samples with normal karyotype, respectively. The samples of the GSE12417-GPL570 subset divide into 23 samples with and 56 samples without FLT3 ITD mutation while the GSE12417-GPL96 subset contains 81 FLT3 ITD and 82 FLT3 WT samples. The gene expression cohort by Verhaak contains 187 samples with normal karyotype out of which 78 were FLT3 ITD mutated and 109 were FLT3 WT. Finally, the dataset by Valk includes 103 normal karyotype samples, which contained 45 FLT3 ITD mutated and 58 FLT3 WT samples. 

### 2.2. Differential Expression Analysis

The samples of the Valk [28], Verhaak [29], and the Metzeler [26] dataset were split according to their FLT3 ITD status into a FLT3 ITD mutated and FLT3 WT sample group. Genes were tested for differential expression in FLT3 ITD mutated samples compared to FLT3 WT samples by performing a two-sided Student’s *t*-test followed by multiple-testing correction using the Benjamini–Hochberg method. Genes with an adjusted *p*-value of at most 0.05 were considered as significantly differentially expressed. Genes with an adjusted *p*-value between 0.05 and 0.1 were marked as not significant with a trend towards upregulation in case of a positive t-value or downregulation if the t-value was negative. The analysis was performed using the statistic software package R v3.6.3 (www.r-project.org).

### 2.3. Expression-Dependent Survival Analysis

AML samples of the Valk [28], Verhaak [29], and the Metzeler [26] dataset were filtered for normal karyotype samples and split according to their FLT3 ITD status into FLT3 ITD mutated and FLT3 WT samples. The expression of the candidate genes was assessed using matching probe-set IDs according to the manufacturer’s array annotation. For each candidate gene, FLT3 ITD mutated and FLT3 WT samples were grouped according to their expression level into high- and low-expressing samples. Cut-offs between the 15th and the 85th percentile of gene expression values, incremented in steps of 1, were tested to obtain the optimal separating cut-off value. Survival analysis (overall survival) was performed by applying the Kaplan–Meier estimator from the Survival Analysis R-package v3.2-7 [30]. To test for significantly different survival between high and low gene expressing samples, a log-rank test war performed. A *p*-value of at most 0.05 was considered to be significant. In case of multiple cut-offs with a significant *p*-value, the cut-off closest to the 50th percentile was chosen.

### 2.4. Cell Lines

RAW264.7 and MC3T3-E1 cells, widely used for studying bone cells, were used to establish an OC and OB model that can be used to analyse the influence of FLT3 ITD on gene expression. The macrophage precursor cell line RAW264.7 and osteoblastic cell line MC3T3-E1 were kindly provided by Lorena Tuchscherr de Hauschopp (Jena University Hospital) and were maintained at 37 °C in a humified atmosphere of 5% CO_2_. RAW264.7 cells were cultivated in high glucose DMEM medium supplemented with 10% FCS, and MC3T3-E1 cells were cultivated in alpha MEM containing 10% FCS. Cells were stably transduced three times with pseudotyped retroviral particles expressing respective FLT3 WT or FLT3 ITD GFP constructs [31] in the presence of 8 μg/mL polybrene (1,5-dimethyl-1,5- diazaundecamethylene polymethobromide, Sigma-Aldrich, Deisenhofen, Germany). Cell populations stably expressing Flt3 were sorted for similar receptor levels using bicistronically expressed GFP using the BD AIRA sorting system (Becton Dickinson, Heidelberg, Germany).

### 2.5. Gene Expression Analysis in Cell Lines on Selected Genes

RNA was extracted from RAW264.7 cells and MC3T3-E1 cells, respectively, using a NucleoSpin RNA Mini Kit (Macherey-Nagel, Düren, Germany) according to the manufacturer’s instructions. RNA was used for cDNA synthesis using the First Strand cDNA synthesis Kit (Thermo Scientific, Bonn, Germany). cDNA corresponding to 50 ng RNA was used for qPCR analysis. Primers for amplification of transcripts (listed in the Supplementary Materials Table S1) were purchased from Sigma-Aldrich, Deisenhofen, Germany. Real-time qPCR reactions were carried out using the QuantStudio 3 Real-Time PCR System (Thermo Scientific, Bonn, Germany) using a Maxima SYBR Green/ROX qPCR master Mix (Thermo Scientific, Bonn, Germany). After initial denaturation for 10 min at 95 °C, 45 cycles of 95 °C for 15 s, 62 °C for 30 s and 72 °C for 30 s were performed, followed by 15 s at 95 °C, 1 min at 60 °C, and 15 s at 95 °C. For quantification of gene expression, double delta Ct analysis was used. ΔCt was determined to the housekeeping gene beta-Actin. Relative expression was calculated in relation to the transduced parental RAW264.7 cell line. Primers for amplification of transcripts (see Supplementary Materials Table S1) were purchased from Sigma-Aldrich, Deisenhofen, Germany. Statistical significances were calculated using ANOVA and PostHoc test. The analysis was accomplished in RStudio v1.3.1093.

## 3. Results

### 3.1. Differentially Expressed Genes (DEG) in FLT3 ITD AML

The recently observed effects of mutant FLT3 on bone structures [24] raised the question of which molecular mechanisms control the aberrant bone formation. The identification of differentially expressed genes (DEGs) encoding cytokines contributing to bone homeostasis of AML patients with mutant FLT3 provides the first insights into the signalling networks controlling the differentiation and activity of cell types contributing to the formation of the haematopoietic niche/bone homeostasis. To analyse the impact of oncogenic FLT3 ITD signalling on gene expression, clustering of data derived from AML FLT3 ITD patient samples and AML FLT3 WT patient samples was performed. 

**Figure 1:**
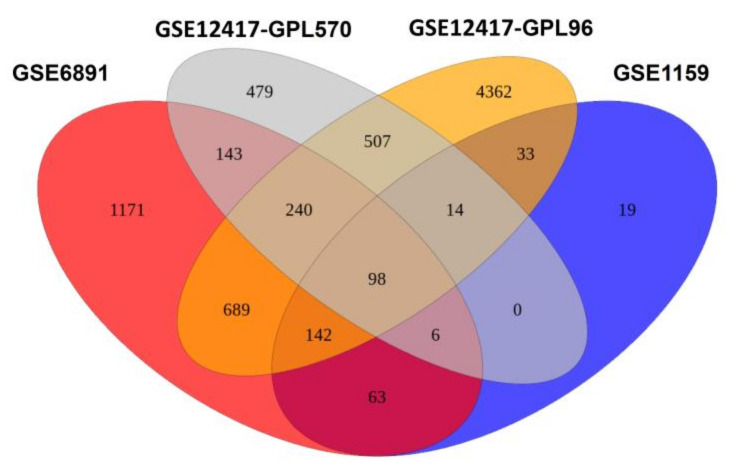
Venn diagram of differentially expressed genes (DEGs) of FLT3 ITD-positive AML samples compared to FLT3 WT AML patient samples in data sets from Verhaak, Valk, and Metzeler. The Venn diagram elucidates the number of genes, which are differentially expressed in the indicated data sets. Red: Verhaak data set GSE6891; grey: Metzeler data set GSE12417-GPL570; orange: Metzeler data set GSE12417-GPL96; blue: Valk data set GSE1159.

DEGs were obtained from four data sets available at the Gene Expression Omnibus (GEO) repository at the National Center for Biotechnology Information. The data are based on transcriptome-profiling microarrays of blasts and mononuclear cells from BM of normal karyotype AML patients. DEG were assembled by comparing AML samples carrying mutated FLT3 ITD to AML samples encoding FLT3 WT alleles. The data sets showed different numbers of significantly up- or downregulated genes in response to the occurrence of FLT3 ITD (Supplementary Materials Table S2). The amount of DEG of particular AML patient cohorts is summarized in Figure 1.

Table 1 provides the information about the DEG-encoding components controlling osteogenesis. While some components did not show clear trends for up- or downregulation in response to the mutational status of FLT3 in the various data sets, the expression of several genes was significantly altered in FLT3 ITD-positive AML samples in individual data sets.

**Figure 2:**
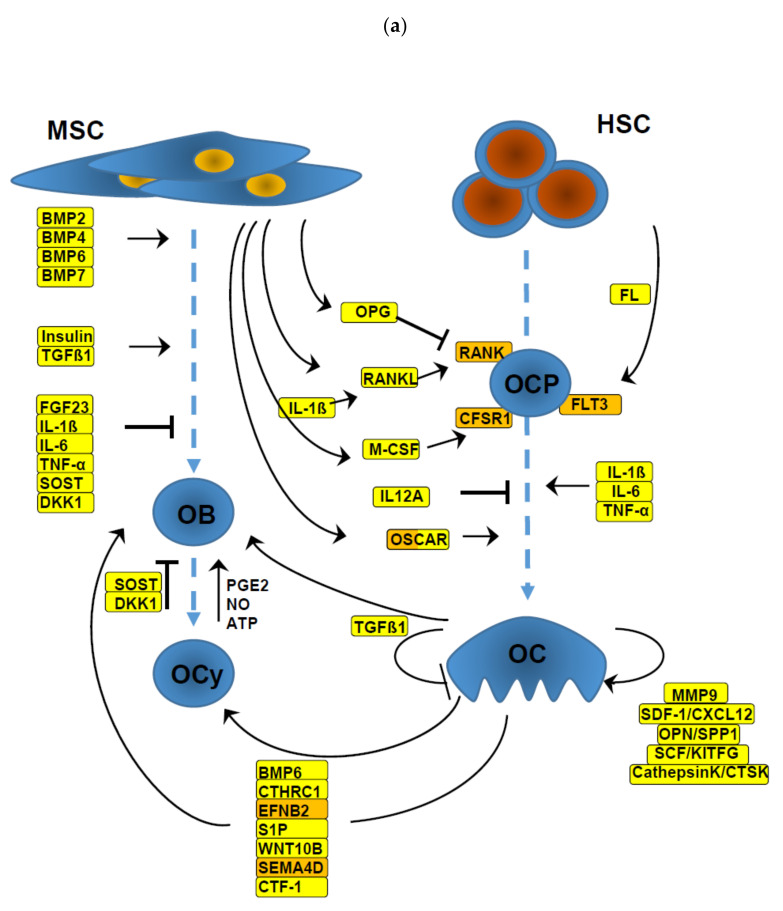

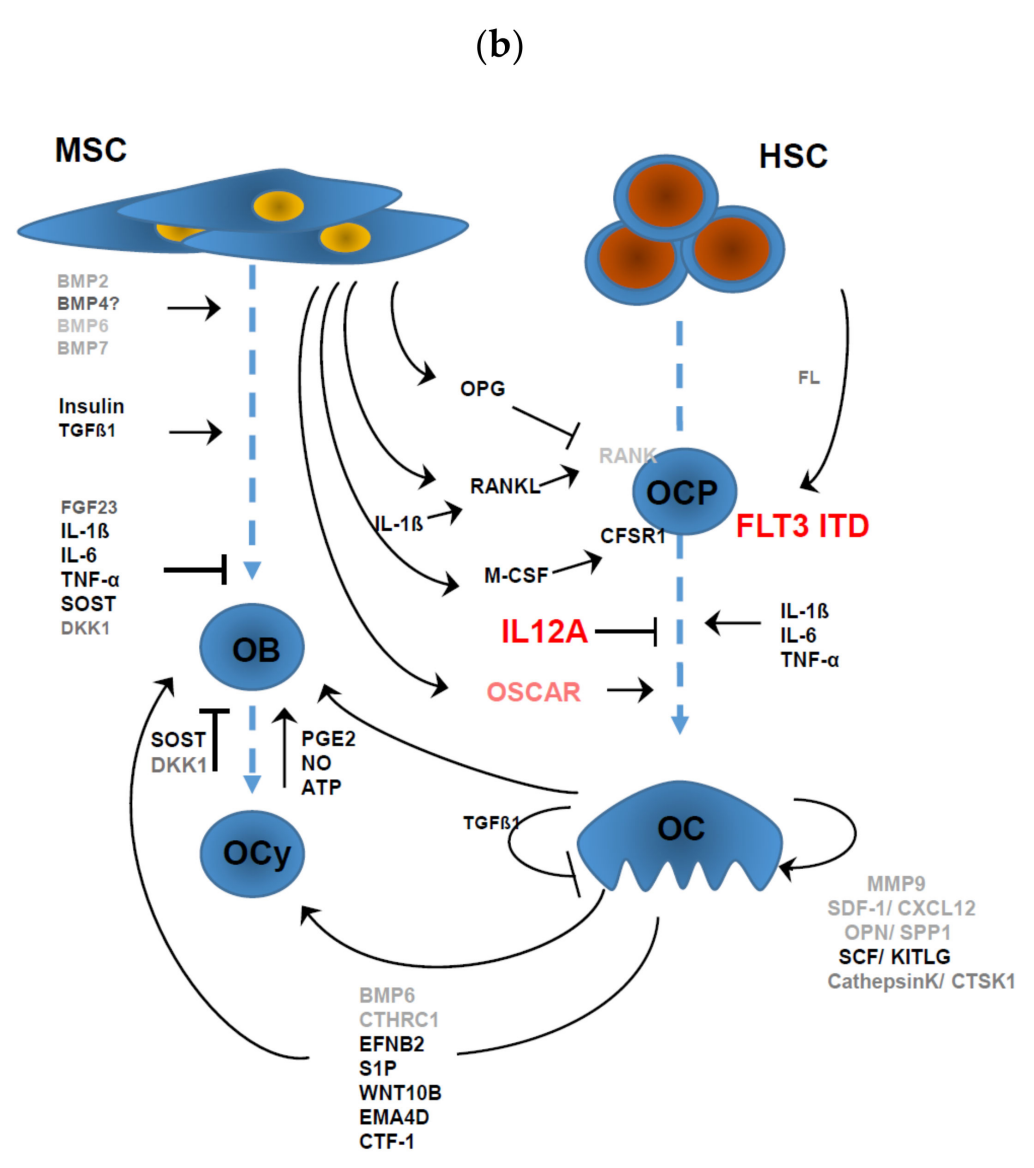
Cytokine network controlling bone homeostasis. The schematic depicts the main components involved in differentiation of osteoblasts (OBs) and osteocytes (OCys) derived from mesenchymal stem cells (MSCs) as well as the differentiation of osteoclasts (OCs) derived from haematopoietic stem cells (HSCs) maturated via OC precursor cells (OCPs). (**a**) Secretory components are framed in yellow, membrane-localized components are indicated in orange. Arrows indicate stimulatory, blocked symbols indicate inhibitory activities. (**b**) Overview about differentially expressed genes (DEGs) of FLT3 ITD-positive compared to FLT3 WT AML patient samples. Upregulated genes are marked in red (significantly up) or rose (trend of upregulation). Downregulated genes are presented in grey.

We addressed the question of which cytokine network components controlling osteoclastogenesis and the formation of OB (summarized in Figure 2a) are affected by FLT3 ITD in blasts and mononuclear cells of AML patients.

To illustrate the effect of FLT3 ITD on their gene expression, Figure 2b provides an overview of components with altered DEGs. FLT3 ITD-positive samples showed a strong upregulation of the FLT3 receptor compared to FLT3 WT samples (Table 1, Figure 2b). *IL12α*, required for T-cell-dependent induction of interferon gamma [32], was upregulated in FLT3 ITD samples with a high significance and showed a consistent increase of 3–6-fold compared to FLT3 WT samples in all data sets.

### 3.2. FLT3 ITD Affects Osteoclastogenesis of HSCs

RANKL and M-CSF secreted by mesenchymal cells, including bone-marrow stromal cells, OB, and osteocytes, drives OC differentiation. Despite being insignificant, *M-CSF* and RANKL receptor *RANK* were shown to be preferentially downregulated in FLT3 ITD-positive AML samples. Upon depletion of M-CSF, the FLT3 ligand FL can compensate its role in osteoclastogenesis [17]. Like M-CSF, FL was predominately downregulated in FLT3 ITD-positive AML samples. The costimulatory receptor OSCAR also promotes OC differentiation through activation of NFATc1 [33]. In contrast to M-CSF and RANK, *OSCAR* showed a trend towards increased expression in FLT3 ITD-positive AML samples. As already indicated above, *IL12α*, known to inhibit osteoclastogenesis [34,35], was significantly upregulated in the presence of FLT3 ITD in all patient cohorts. Thus, osteoclastogenesis appears to be reduced in FLT3 ITD-positive patients. Consequently, *MMP9* and *CXCL12*, secreted by mature OC, were found to be downregulated in the FLT3 ITD AML patients. Taken together, these data indicate that the formation of OC is hampered by downregulation of the osteoclastogenesis-promoting signalling routes and the upregulation of *IL12α*. Since osteopontin (OPN, encoded by *SPP1*) controls mobilization of OCs and is therefore actively involved in bone resorption, its reduced expression in FLT3 ITD samples, which is significant in the Valk dataset, might explain the increased formation of trabeculae observed in FLT3 ITD mice [24].

### 3.3. FL Induced FLT3 Signalling Regulates Cytokines

In knockout FL or FLT3 rheumatoid arthritis mouse models, increased OC formation and bone damage have been observed [36]. Thus, the FL-FLT3 signalling route appears to be a crucial factor responsible for dendritic cell development and osteoclastogenesis in mouse bone marrow. In addition, these mice were reported to have reduced numbers, not only of dendritic cells but also neutrophils, NK cells, and monocytes and therefore, a reduced pool of OC precursors. By promoting transcription of *interferon regulatory factor 8* (*IRF8*) and sustaining a balance between protective regulatory T cells and pathogenic T helper 17, FL may serve as a negative regulator of OC development. Thus, Voronov and co-workers concluded a pro- and anti-resorptive function of FL [21]. In FLT3 ITD-positive AML samples, a trend towards downregulation of FL was observed. 

### 3.4. Effect of FLT3 ITD on Regulation of OB Differentiation

While OB and their precursors affect osteoclastogenesis, OC-derived factors influence the differentiation and activities of OB and osteocytes (recently reviewed in [37]). Bone morphogenic protein (BMP) superfamily members, which have fundamental roles in both embryonic skeletal development and exhibit high osteogenic activity [38], were all downregulated in FLT3 ITD-positive AML patients, with a significant downregulation in the Metzeler dataset of *BMP2* and *BMP7*. By crucially contributing in the crosstalk of OC and OB, the sphingolipid machinery has a pivotal role in bone homeostasis [39]. Sphingosine-1-phosphate (S1P) and its cognate receptor S1PR1 are known as key players in osteoimmunology [40]. Thus, preferential downregulation of *S1PR1* indicates reduced osteoimmunological response in FLT3 ITD AML samples. DKK1, which is secreted from mature OBs, implements a negative feedback to Wnt/β-catenin signalling of OB precursor cells and therefore downregulates OB formation [41,42]. Expression of *DKK1* was downregulated in FLT3 ITD-positive AML samples, although only in the Metzeler GSE12417-GPL96 data set was this significant. Likewise, DKK1 co-receptor Kremen2 [43] showed a reduced expression. The expression of *fibroblast growth factor* (*FGF*) *23*, which also negatively regulates OB differentiation and matrix mineralization [44], was significantly reduced in the Metzeler data set GSE12417-GPL96. 

### 3.5. Gene Expression in OC and OB Precursor Cell Lines in Response to FLT3 ITD or FLT3 WT Expression

In order to analyse if the expression of Flt3 ITD has consequences on the gene expression in bone cells, the OC precursor cell line RAW264.7 and OB precursor cell line MC3T3-E1 were stably transduced with retroviral expression constructs encoding murine Flt3 WT or Flt3 ITD [31]. Gene expression of components involved in osteoclastogenesis was selectively influenced by induction of Flt3 WT or Flt3 ITD proteins. The tested genes are listed in Supplementary Materials Table S1. Interestingly, in RAW264.7 cells, an elevated expression of *RANK* and *RANKL* in the presence of Flt3 ITD indicates increased signalling along this axis in the presence of Flt3 ITD (Table 2a). While the secretion of Cxcl12 remained unchanged in Flt3 ITD-expressing cells, secretory *Mmp9* and *Spp1* were downregulated, indicating a tendency for reduced activity of Flt3 ITD expressing OC precursor cells. Similar to FLT3 ITD AML patient data, the expression of *Oscar* was upregulated in RAW264.7 Flt3 ITD cells. 

In the OB precursor cell line MC3T3-E1, the expression of bone morphogenetic proteins Bmp4 and Bmp6 were downregulated in Flt3 ITD-expressing cells compared to the untransduced parental cell line. As the opposite, Bmp2 showed a more than 3-fold expression increase. However, in Flt3 WT-expressing cells, the expression of *Bmp2*, *Bmp4*, and *Bmp6* all showed a trend towards upregulation, indicating a pro-osteoblastic effect. *Opg*, which acts as a decoy for Rankl and therefore prevents its binding to the Rank receptor [12], was elevated in MC3T3-E1 Flt3 ITD cells; Flt3 WT cells suppressed its expression. Expression of *Sost* as a negative regulator of bone growth and formation was elevated in the Flt3 ITD-producing cell line while the expression in Flt3 WT cells remained almost unchanged. Moreover, Flt3 ITD overexpression in MC3T3-E1 cells resulted in a 9-fold induction of *Fgf23*, which was also shown to suppress OB differentiation and matrix mineralization [44]. Taken together, Flt3 ITD-mediated effects point to an inhibitory effect of Flt3 on the formation of OB in MC3T3-E1 cells.

### 3.6. Survival Prognosis of AML Patients Based on the Expression of Genes Involved in Bone Homeostasis

Factors involved in bone homeostasis frequently affect leukaemogenesis. Thus, in addition to detecting DEGs, we addressed the question if the expression level of factors controlling bone homeostasis affects the survival of AML patients. Patient prognosis was selectively calculated for FLT3 ITD, FLT3 WT, as well as all AML patients independent of the mutational status of FLT3. Supplementary Materials Table S3 summarizes the prognostic survival separately obtained from data sets from Verhaak (GSE6891), Valk (GSE1159), and Metzeler (GSE12417-GPL570 and GSE12417-GPL96).

**Figure 3:**
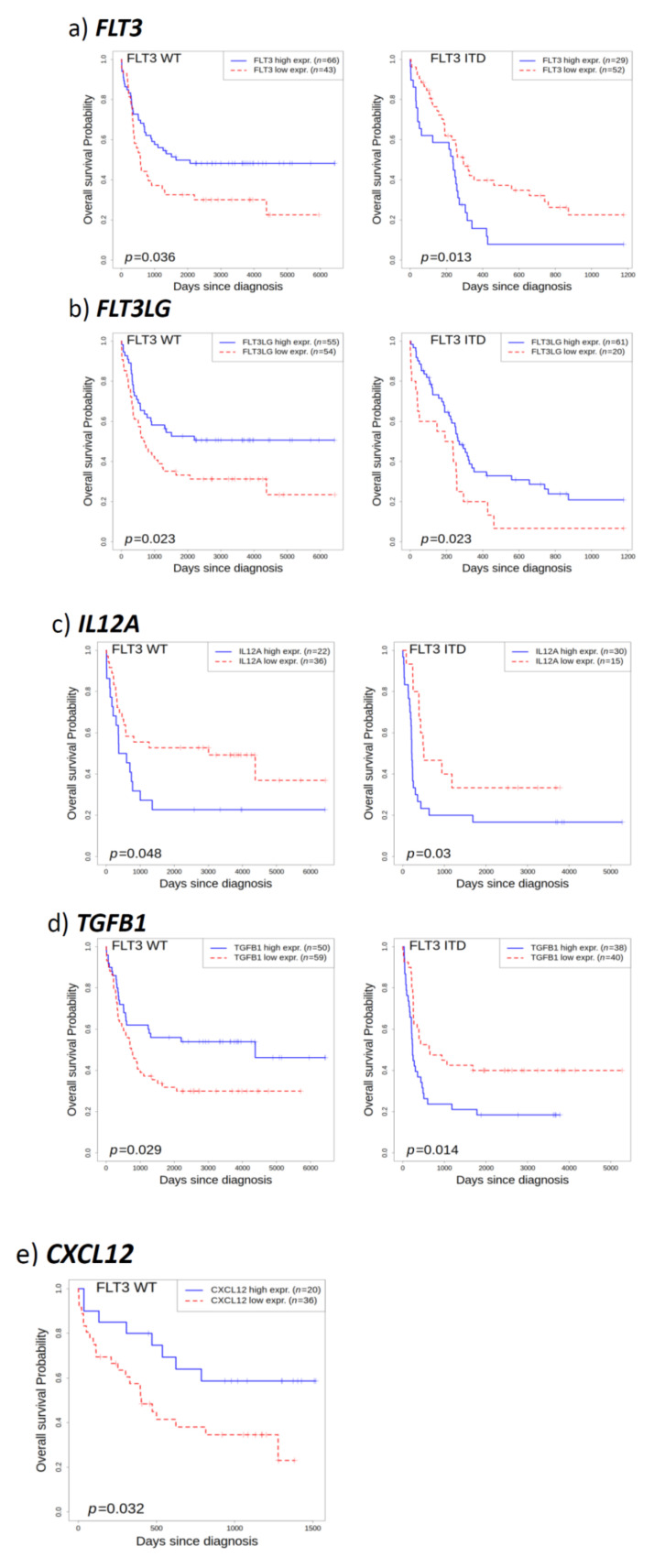
Prognosis of AML patients based on gene expression. Overall survival of FLT3 WT AML patients and FLT3 ITD AML patients with low (red, dotted) and high (blue) expression of indicated genes. Patient survival in response to expression of FLT3 (**a**) based on data set GSE6891 (for FLT3 WT) or GSE12417-GPL96 (for FLT3 ITD), FLT3LG (**b**) based on data set GSE6891 (for FLT3 WT) or GSE12417-GPL96 (for FLT3 ITD), IL12A (**c**), based on data set GSE1159, TGFB1 (**d**) based on data set GSE6891, or CXCL12 (**e**) based on data set GSE12417-GPL570. Significance values (*p* values) are given in the graphs.

Increased expression of mutant FLT3 ITD was detrimental for patient survival, although statistical significance for this effect was only noted in the GSE12417-GPL96 dataset (Figure 3a). Intriguingly, FLT3 WT AML patients have a better prognosis if they produce high levels of FLT3 (Supplementary Materials Table S3, Figure 3a). Surprisingly, AML patients with high FL production have a trend of better prognosis, irrespective of the mutational status of FLT3 (Supplementary Materials Table S3a–c, Figure 3b).

High expression levels of *IL12α* have a negative impact on patient survival, irrespective of the mutational status of FTL3 (Supplementary Materials Table S3, Figure 3c) while high *IL12α* levels resulted in significantly reduced survival in data sets integrating all AML patients. Similarly, high *TGF-β1* (transforming growth factor-β1, encoded by *TGFB1)* levels resulted in significantly worse outcomes for AML patients in two data sets. Selective calculation revealed that for AML patients expressing FLT3 WT, high expression of *TGF-β1* was associated with a better prognosis, while it was mainly detrimental in FLT3 ITD-positive AML (Supplementary Materials Table S3, Figure 3d). It has recently been demonstrated that AML transforms the bone marrow niche into a leukaemia-permissive microenvironment through exosome secretion of CXCL12 [45]. Low expression of *CXCL12* was found in patients with a better outcome in FLT3 WT samples (Supplementary Materials Table S3, Figure 3e). Other factors did not consistently or only insignificantly affected patient survival. Taken together, patient survival data indicate that the gain of osteoclastogenesis promoting components is inversely correlated to AML patient survival.

## 4. Discussion

Oncogenic FLT3 ITD mutations commonly found in AML patients have a great impact on gene expression. Aberrant gene expression in FLT3 ITD-positive AML patients is a known feature of this disease [3,46,47,48]. The molecular mechanisms of how oncogenic mutant FLT3 controls aberrant leukaemogenesis have been frequently addressed (for a recent review, see [3]). Since mutant FLT3 also affects the BM microenvironment [2,24], we focused here on the regulation of the cytokine network controlling bone homeostasis. This is of particular importance, since the BM niche affects the development of leukaemic stem cells and therefore affects the minimal residual disease and relapse of AML patients [2,49]. The aim of this study was to elucidate the effect of FLT3 ITD on the expression of genes controlling osteoclastogenesis, the formation and activity of OB, and to identify factors that are crucial for the prognosis of AML patients. These data will help to find prognostic factors that participate in the development of the haematologic disease.

Figure 2a illustrates the primary network controlling osteoclastogenesis and the formation of OB. It reveals that the intimate interwoven OC–OB-crosstalk is mediated predominantly by cytokines secreted by these cell types. Their concerted expression results in fine-balanced bone homeostasis. Mutant FLT3 ITD results in aberrant production of factors involved in osteoclastogenesis and formation of OBs (Figure 2b, Table 1). In particular, OC precursor-stimulating signalling pathways like RANKL-RANK, M-CSF-CSFR1, and FL-FLT3 seem to be negatively affected by suppressed cytokine (FL) or receptor (RANK, CSFR1) production. In addition, IL12α, which inhibits osteoclastogenesis, was heavily overexpressed in FLT3 ITD-positive AML samples. Consequently, MMP9, which plays an important role in degradation of the extracellular matrix and OC precursor recruitment [13,15], and stromal cell-derived factor-1 (SDF-1, encoded by *CXCL12*), which acts as a key factor in the HSC niche [50], are downregulated in FLT3 ITD AML. This alteration indicates a possible reduction of OC activity at the haematopoietic niche. Taken together, osteoclastogenesis seems to be negatively controlled by FLT3 ITD. Forced expression of FLT3 ITD in the OC precursor cell line RAW264.7 resulted in reduced *MMP9* expression, validating the findings in AML patient samples.

In analogy, OB differentiation appears to be negatively affected in FLT3 ITD-positive AML patients. Importantly, OC-derived coupling factors BMP2, BMP6, and BMP7, which stimulate bone formation by recruiting osteoprogenitors to the sites of bone remodelling [37,51], were downregulated in FLT3 ITD-positive AML patients. In addition, the receptor S1PR1 receiving OB stimulatory signals by S1P secreted from OC was also drastically reduced in FLT3 ITD-positive AML samples compared to FLT3 WT samples. The enhanced *TGF-β1* expression in the FLT3 ITD AML background might have an ambivalent effect on bone homeostasis. TGF-β1 is known to induce OC apoptosis [52,53,54] but in turn promotes the migration of mesenchymal stem cells to the bone remodelling sites to couple bone resorption and formation [55]. Thus, elevated *TGF-β1* levels might result in imbalanced OB activity beside the presence and activity of OC. The controversial effects of TGF-β1 on the overall survival of FLT3 WT and FLT3 ITD AML patients corroborate the observation that the dysregulation of TGF-β1 causes clinically relevant skeletal disorders. OPN was shown to negatively regulate and limit the number of endosteal stem cells [55,56]. Moreover, the motility and bone resorption of metaphyseal trabeculae is controlled by OPN [57]. Thus, the FLT3 ITD-mediated suppression of *SPP1* might result in enhanced OC motility and capacity of bone resorption. Poor prognosis of AML patients with high *SPP1* expression supports the hypothesis that abberrant osteoclastogenesis promotes the haematological aberration. Since the main role of the stem cell niche component SCF (encoded by *KITLG*) is the maintenance of HSC and their retention in the BM niche [49], its enhanced level might stimulate leukaemogenesis.

In summary, the analysis of DEG of AML patients suggests that FLT3 ITD does not only control development of aberrant haematopoiesis but has an impact on BM niche formation for clonal development of FLT3 ITD-positive blasts. Altered signalling networks of cell types involved in bone homeostasis have effects on the regulation of niche formation embedding the development of leukaemic stem cells. Thus, insight in the mutant FLT3-mediated impact on processes controlling bone homeostasis is the prerequisite to further understand leukaemogenesis.

The survival differences in groups characterized by different expression levels of genes suggest that genes controlling osteoclastogenesis or OB formation and activity might be clinically relevant. Clearly, further clinical validation is required to corroborate these concepts.

## Figures and Tables

**Table 1:** FLT3 ITD affects the expression of genes involved in bone homeostasis. Differences in gene expression of FLT3 ITD positive to FLT3 WT AML patient samples were analysed using data AML data sets from Verhaak (GSE6891), Valk (GSE1159), and Metzeler (GSE12417-GPL570 and GSE12417-GPL96). Differential gene expression is presented with adjusted p-values. Significance was set at *p* < 0.05. Stars indicate as follows * *p* < 0.05; ** *p* < 0.01, *** *p* < 0.001, **** *p* < 0.0001; n.d., not determined.

Gene	Probe	GSE1159	GSE6891	GSE12417-GLP96	GSE12417-GPL570
**BMP2**	205289_at	−1.45, *p* = 0.52	−2.03 *p* = 0.24	* −2.42, *p* = 0.049	−1.66, *p* = 0.33
	205290_s_at	−2.70, *p* = 0.14	−1.86, *p* = 0.30	−2.40, *p* = 0.051	−1.87, *p* = 0.27
**BMP6**	206176_at	−2.04, *p* = 0.30	−2.46, *p* = 0.12	−1.0, *p* = 0.44	−2.27, *p* = 0.16
	215042_at	0.25, *p* = 0.94	−0.34, *p* = 0.88	−1.22, *p* = 0.34	−1.51, *p* = 0.38
**BMP7**	209590_at	−0.76, *p* = 0.78	−1.67, *p* = 0.37	* −3.81, *p* = 0.02	−0.51, *p* = 0.8
	209591_s_at	0.50, *p* = 0.86	0.99, *p* = 0.61	−1.92, *p* = 0.12	0.56, *p* = 0.78
**CSF1** **(M-CSF)**	209716_at	−0.10, *p* = 0.98	1.12, *p* = 0.59	1.88, *p* = 0.13	1.04, *p* = 0.59
	210557_x_at	−0.88, *p* = 0.74	−0.04, *p* = 0.99	−1.52, *p* = 0.23	−0.11, *p* = 0.7964
	211839_s_at	−0.89, *p* = 0.74	0.04, *p* = 0.98	n.d.	−0.77, *p* = 0.7694
	207082_at	−0.59, *p* = 0.84	−0.038, *p* = 0.99	−0.62, *p* = 0.62	0.54, *p* = 0.79
**CSFR1**	203104:at	−1.85, *p* = 0.37	−0.74, *p* = 0.72	* 2.66, *p* = 0.03	0.41, *p* = 0.58
**CTHRC1**	225681_at	n.d.	−1.63, *p* = 0.37	n.d.	−2.23, *p* = 0.17
**CXCL12**	203666_at	−1.16, *p* = 0.63	−1.71, *p* = 0.34	* −2.42, *p* = 0.048	−0.28, *p* = 0.89
	209687_at	−1.66, *p* = 0.43	−1.33, *p* = 0.50	−1.48, *p* = 0.23	−0.51, *p* = 0.80
**DKK1**	204602_at	−0.0036, *p* = 0.99	−1.86, *p* = 0.29	* −3.02, *p* = 0.013	0.85, *p* = 0.65
**FGF23**	221166_at	−0.88, *p* = 0.74	−1.64, *p* = 0.37	** −3.55, *p* = 0.004	−0.90, *p* = 0.64
**FLT3**	206674_at	3.32, *p* = 0.057	*** 4.67, *p* = 0.00066	** 3.66, *p* = 0.0029	3.27, 0.05
**FLT3LG**	206980_s_at	−2.21, *p* = 0.25	−1.82, *p* = 0.30	0.34, *p* = 0.81	−1.77, *p* = 0.29
	210607_at	−2.17, *p* = 0.26	* −2.99, *p* = 0.045	−0.03, *p* = 0.98	−2.31, *p* = 0.15
**IL12A**	207160_at	* 3.53, *p* = 0.044	*** 5.22, *p* = 0.00013	**** 6.38, *p* = 1.49e-6	3.32, *p* = 0.05
**IL-1β**	39402_at	−0.53, *p* = 0.86	−0.67, *p* = 0.75	1.45, *p* = 0.25	0.83, *p* = 0.66
**KITLG (SCF)**	207029_a	0.45, *p* = 0.87	1.37, *p* = 0.49	−0.73, *p* = 0.59	0.15, *p* = 0.95
	211124_s_a	1.22, *p* = 0.62	2.61, *p* = 0.10	−0.63, *p* = 0.64	0.36, *p* = 0.87
**Kremen2**	219692_at	−1.0, *p* = 0.70	−0.81, *p* = 0.69	−2.00, *p* = 0.11	0.85, *p* = 0.65
**MMP9**	203936_s_at	−1.26, *p* = 0.59	−2.65, *p* = 0.08	−2.26, *p* = 0.06	−1.59, *p* = 0.35
**OSCAR**	1554503_a_at	n.d.	1.49, *p* = 0.43	n.d.	1.82, *p* = 0.27
**Runx2**	216994_s_at	1.06, *p* = 0.67	−0.88, *p* = 0.66	−0.55, *p* = 0.67	−0.67, *p* = 0.74
	221282_x_at	0.42, *p* = 0.90	−1.16, *p* = 0.57	−0.09, *p* = 0.96	1.06, *p* = 0.57
	221283_at	0.39, *p* = 0.90	−2.08, *p* = 0.22	−2.93, *p* = 0.169	−0.76, *p* = 0.72
	236858_s_at	n.d.	−1.44, *p* = 0.46	1.58, *p* = 0.36	n.d.
	236859_at	n.d.	−1.83, *p* = 0.30	−2.81, *p* = 0.96	n.d.
**S1PR1**	204642_at	−3.0, *p* = 0.09	* −3.36, *p* = 0.02	0.79, *p* = 0.55	−1.92, *p* = 0.25
**SPP1**	209875_s_at	−2.11, *p* = 0.28	* −3.30, *p* = 0.02	−1.64, *p* = 0.19	−1.79, *p* = 0.28
**TGFβ1**	203084_at	−1.87, *p* = 0.36	−0.10, *p* = 0.96	0.72, *p* = 0.59	−1.97, *p* = 0.23
	203085_s_at	−0.52, *p* = 0.86	0.38, *p* = 0.87	* 3.05, *p* = 0.012	2.63, *p* = 0.11
**TNFα**	207113_s_at	0.33, *p* = 0.92	−0.27, *p* = 0.91	0.61, *p* = 0.65	0.47, *p* = 0.82
**TNFRSF11A** **(RANK)**	207037_at	−0.84, *p* = 0.75	−1.63, *p* = 0.38	−0.62, *p* = 0.64	0.67, *p* = 0.73
	238846_at	n.d.	−2.69, *p* = 0.08	n.d.	0.86, *p* = 0.65
**TNFRSF11B** **(OPG)**	204932_at	−1.0, *p* = 0.69	−1.13, *p* = 0.58	0.77, *p* = 0.56	0.55, *p* = 0.79
	204933_s_at	1.49, *p* = 0.50	−0.35, *p* = 0.88	−0.49, *p* = 0.72	−0.20, *p* = 0.92
**TNFSF11** **(RANKL)**	210643_at	0.48, *p* = 0.88	1.00, *p* = 0.60	2.20, *p* = 0.075	−0.45, *p* = 0.83

**Table:** (**a**) Gene Expression in RAW264.7.

Gene	FLT3 WT		FLT3 ITD	
*Cxcl12* (Sdf-1)	0.65	*p* = 0.4009	1.06	*p* = 0.9683
*Mmp9*	0.86	*p* = 0.1168	0.38	*p* = 0.0863
*Oscar*	0.71	*p* = 0.8497	1.81	*p* = 0.3517
*Spp1*	0.28	*p* = 0.0464	0.45	*p* = 0.1204
*Tnfrsf11a* (Rank)	0.54	*p* = 0.7416	2.14	*p* = 0.2281
*Tnfsf11* (Rankl)	0.51	*p* = 0.3386	1.80	*p* = 0.1015

**Table:** (**b**) Gene Expression in MC3T3-E1.

Gene	FLT3 WT		FLT3 ITD	
*Bmp2*	1.48	*p* = 0.6012	3.39	*p* = 0.9330
*Bmp4*	1.74	*p* = 0.3289	0.57	*p* = 0.6563
*Bmp6*	2.58	*p* = 0.4529	0.68	*p* = 0.9636
*Dkk1*	1.27	*p* = 0.7421	3.32	*p* = 0.6392
*Fgf23*	0.90	*p* = 0.9980	8.95	*p* = 0.0073
*Runx2*	0.90	*p* = 0.9824	1.03	*p* = 0.9983
*Sost*	1.27	*p* = 0.9855	3.32	*p* = 0.3969
*Tnfrsf11b* (Opg)	0.11	*p* = 0.4735	2.60	*p* = 0.3441

## Supplementary Materials

The following are available online at https://www.mdpi.com/2073-4409/9/11/2443/s1, Table S1: Primers of genes for qRT-PCR in murine RAW 267.4 and MC3T3 E1 cells; Table S2: Significantly DEG of individual AML data sets; Table S3: Prognostic risc score for AML patients.
