# Supplementary material for: Aberrant Bone Homeostasis in AML Is Associated with Activated Oncogenic FLT3-Dependent Cytokine Networks
Source: Cells. 2020 Nov 9;9(11):2443. doi: 10.3390/cells9112443 (PMC7697865; doi:10.3390/cells9112443)
Supplement: Supplementary file 1 [file cells-09-02443-s001.pdf]

cells MDPI

Supplementary Table S1. Primers of genes for qRT-PCR in murine RAW267.4 and MC3T3-E1 cells.

Gene	Forward Primer (5′-3′)	Reverse Primer (5′-3′)
Bmp2	CGCTCCACAAACGAGAAAAG	CAGTCATTCCACCCCACATC
Bmp4	CTCAAGGGAGTGGAGATTGG	CTTCTGCGGGTCAAGGTATG
Bmp6	TAGCAATCTGTGGGTGGTGA	GAAGGGCTGCTTGTCGTAAG
Cathepsin K	CTCGGCGTTTAATTTGGGAGA	TCGAGAGGGAGGTATTCTGAGT
Cxcl12	GGAGGATAGATGTGCTCTGG AAC	AGTGAGGATGGAGACCGTGGT G
Dkk1	CAGTGCCACCTTGAACTCAGT	CCGCCCTCATAGAGAACTCC
Dmp1	TGTCATTCTCCTTGTGTTCCTT TG	AGAGCTTTCAGATTCAGTATTG TCGTAT
Fgf23	ATGCTAGGGACCTGCCTTAGA	GGAGCCAAGCAATGGGGAA
Flt3lg	AGAGGAGACACCAAGACAGA AA	CCACGTCTATGTTTTGCAGGT
Gapdh	CAGGTTGTCTCCTGCGACTT	CCCTGTTGCTGTAGCCGTA
Mmp9	GCTGACTACGATAAGGACGG CA	TAGTGGTGCAGGCAGAGTAGG A
Nfatc1	GTTCCTTCAGCCAATCATCC	GGAGGTGATCTCGATTCTCG
NFκB	GGAACCGAACTACCCACCTTT	GCCCAGATCCACTGAATCCA
Tnfrsf11b (Opg)	CCTTGCCCTGACCACTCTTAT	CACACACTCGGTTGTGGGT
Oscar	TCTGCCCCCTATGTGCTATC	CTCCTGCTGTGCCAATCAC
Tnfrsf11a (Rank)	CCAGGAGAGGCATTATGAGC A	ACTGTCGGAGGTAGGAGTGC
Tnfsf11 (Rankl)	AGCCGAGACTACGGCAAGTA	AAAGTACAGGAACAGAGCGAT G
Runx2	AGAGTCAGATTACAGATCCC AGG	TGGCTCTTCTTACTGAGAGAGG
Sost	CGTGCCTCATCTGCCTACTT	TGACCTCTGTGGCATCATTC
Ssp1	GCTTGGCTTATGGACTGAGGT C	CCTTAGACTCACCGCTCTTCAT G
Tgfβ1	AGGGCTACCATGCCAACTTC	CCACGTAGTAGACGATGGGC
Tgfβ1i1	ATGTCACGGTTAGGGGCTC	GGCTTGCATACTGTGCTGTATA G
Tnfα	CAGGCGGTGCCTATGTCTC	CGATCACCCCGAAGTTCAGTAG
Trap	CACTCCCACCCTGAGATTTGT	CCCCAGAGACATGATGAAGTC A

Supplementary Table S2. Significantly DEG of individual AML data sets.

Dataset	Total Number of AlterdDEG	Number of Upregulated DEG	Number of Downregulated DEG
Metzeler GSE12417-GPL570	1487	884	604
Metzeler GSE12417-GPL96	6085	4378	1788
Valk GSE1159	375	266	109
Verhaak GSE6891	2552	1564	989

Supplementary Table S3. Prognostic risc score for AML patients. Overall survival analysis of AML patients analysed for FLT3 ITD-positive (**a**), FLT3 WT (**b**) or all (**c**) AML patients. Data are derived from data sets GSE1159, GSE6891, GSE12417-GLP96 and GSE12417-GPL570. Tables demonstrate the consequence of high level expression on prognosis. Values indicate the expression cutoffs, which were used to split the patient cohorts in high and low level expressing patients. Significantly ($p < 0.05$) altered prognosis is highlighted in bold and coloured, altered prognosis close to significant ($p < 0.1$) is coloured. Bad prognosis in response to high level expression is highlighted in red; good prognosis is highlighted in green. *p*, *p*-value.; n.s., not significant; n.d., not determined.

(**a**) Prognosis of FLT3 ITD AML patients in response to indicated gene expression.

Gene	Probe	GSE1159	GSE6891	GSE12417-GLP96	GSE12417-GPL570
BMP6	206176_at	**bad (13.27, *p*=0.033)**	n.s.	**bad (8.15, *p*=0.05)**	n.s.
	215042_at	**good (1.6, *p*=0.0001)**	n.s.	**bad (7.8, *p*=0.031)**	n.s.
CTHRC1	225681_at	n d.	n.s.	n.d.	n.s.
CXCL12	203666_at	n.s. trend good (34.7, *p*=0.072)	n.s.	n.s.	n.s.
	209687_at	n.s.	n.s.	n.s.	n.s.
DKK1	204602_at	n.s.	n.s.	**bad (7.07, *p*=0.017)**	n.s.
FLT3	206674_at	n.s.	n.s.	**bad (12.1, *p*=0.013)**	n.s.
FLT3LG	206980_s_at	n.s.	n.s.	**good (7.22, *p*=0.023)**	**good (6.86, *p*=0.02)**
	210607_at	n.s.	n.s.	**good (7.36, *p*=0.046)**	n.s.
IL12A	207160_at	**bad (21.49, *p*=0.03)**	n.s.	n.s.	n.s. trend bad (7.56, *p*=0.058)
MMP9	203936_s_at	n.s.	n.s.	n.s.	n.s.
OSCAR	1554503_a_at	n.d.	n.s. trend bad (7.04, *p*=0.072).	n.d.	n.s. trend bad (7.4, *p*=0.061
SPP1	209875_s_at	n.s.	n.s.	n.s.	
TGFβ1	203084_at	n.s., trend bad (13.7, *p*=0.06)	n.s.	**good (8.37, *p*=0.024)**	n.s.
	203085_s_at	**bad (528.76, *p*=0.03)**	**bad (10.42, *p*=0.014)**	n.s.	n.s.
TNF	207113_s_at	n.s.	**bad (5.81, *p*=0.013)**	n.s.	n.s.
TNFRSF11A	207037_at	**bad (3.73, *p*=0.024)**	n.s.	n.s.	**bad (6.06, *p*=0.017)**
TNFRSF11A	238846_at		n.s.		**bad (5.8, *p*=0.02)**
TNFRSF11B	204932_at	**good (5.38, *p*=0.049)**	n.s.	**bad (5.79, *p*=0.046)**	n.s.
TNFRSF11B	204933_s_at	**bad (1.96, *p*=0.05)**	n.s.	**bad (6.56, *p*=0.049)**	n.s.

(**b**) Prognosis of FLT3 WT AML patients in response to indicated gene expression.

Gene	Probe	GSE1159	GSE6891	GSE12417-GLP96	GSE12417-GPL570
BMP6	206176_at	n.s.	n.s.	n.s.	n.s. trend bad (6.48, *p*=0.072)
	215042_at	n.s.	n.s.	n.s. trend bad (8.16, *p*=0.071)	n.s.
	241141_at	n.d.	n.s.	**bad (7.73, *p*=0.047)**	n.s. trend good (5.86, *p*=0.059)
CTHRC1	225681_at	n.d.	n.s.	n.d.	n.s.
CXCL12	203666_at	n.s.	n.s.	n.s.	**good (7.94, *p*=0.032)**
	209687_at	n.s.	n.s.	n.s.	**good (7.25, *p*=0.034)**
DKK1	204602_at	**good (11.55, *p*=0.037)**	**bad (5.02, *p*=0.025)**	**good (10.86, *p*=0.024)**	n.s.
FLT3	206674_at	**good (565.55, *p*=0.039)**	**good (9.46, *p*=0.036)**	n.s.	n.s.
FLT3LG	206980_s_at	**good (18.56, *p*=0.034)**	n.s.	**good (7.55, *p*=0.049)**	n.s.
	210607_at	n.s.	**good (5.39, *p*=0.023)**	n.s.	n.s. trend good (6.9, *p*=0.056)
IL12A	207160_at	**bad (23.5, *p*=0.048)**	n.s.	bad (7.07, *p*=0.035)	n.s. trend bad (7.23, *p*=0.082)
MMP9	203936_s_at	**bad (55.24, *p*=0.037)**	**bad (6.53, *p*=0.032)**	**good (8.8, *p*=0.02)**	n.s.
OSCAR	1554503_a_at	n.d.	n.s.	n.d.	n.s.
SPP1	209875_s_at	n.s.	n.s.	**bad (7.32, *p*=0.006)**	**bad (6.66, *p*=0.018)**
TGFβ1	203084_at	n.s.	n.s.	**good (8.22, *p*=0.048)**	n.s.
	203085_s_at	**good (731.2, *p*=0.027)**	**good (10.48, *p*=0.029)**	**good (8.22, *p*=0.048)**	n.s.
TNFα	207113_s_at	n.s.	n.s. trend good (5.13, *p*=0.072)	n.s.	n.s.
TNFRSF11A	207037_at	n.s.	n.s.	n.s.	n.s.
	238846_at	n.d.	n.s.	n.s.	n.s.
TNFRSF11B	204932_at	**bad (5.35, *p*=0.02)**	n.s.	n.s.	n.s.
	204933_s_at	n.s.	n.s.	n.s.	**bad (5.67, *p*=0.038)**

(**c**) Prognosis of AML patients in response to indicated gene expression irrespective to the mutational state of FLT3.

Gene	Probe	GSE1159	GSE6891	GSE12417-GLP96	GSE12417-GPL570
BMP6	206176_at	n.s.	n.s.	**bad (8.16, *p*=0.034)**	n.s.
	215042_at	n.s. trend good (2.5, *p*=0.061)	n.s.	**bad (7.83, *p*=0.028)**	n.s.
CTHRC1	225681_at	n.d.	n.s.	n.d.	n.s.
CXCL12	203666_at	n.s. trend good (41.34, *p*=0.053)	n.s. trend good (6.08, *p*=0.089)	**good (9.4, *p*=0.038)**	**good (8.43, *p*=0.011)**
	209687_at	n.s. trend good (3.4, *p*=0.062)	**good (5.05, *p*=0.047)**	n.s.	**good (7.24, *p*=0.035)**
DKK1	204602_at	n.s.	**bad (5.02, *p*=0.025)**	**bad (7.08, *p*=0.045)**	n.s.
FLT3	206674_at	n.s.	**good (10.68, *p*=0.035)**	**bad (12.1, *p*=0.001)**	n.s. trend bad (10.27, *p*=0.09)
FLT3LG	206980_s_at	**good (16, *p*=0.032)**	n.s.	n.s. trend good (7.44, *p*=0.061)	**good (6.87, *p*=0.032)**
	210607_at	n.s.	**good (5.35, *p*=0.003)**	n.s. trend good (7.15, *p*=0.071)	**good (7.0, *p*=0.042)**
IL12A	207160_at	**bad (23.41, *p*=0.002)**	**bad (4.92, *p*=0.004)**	**bad (8.4, *p*=0.004)**	**bad (7.27, *p*=0.036)**
MMP9	203936_s_at	**bad (61.9, *p*=0.035)**	**bad (7.02, *p*=0.029)**	n.s. trend good (8.85, *p*=0.053)	n.s.
OSCAR	1554503_a_at	n.d.	n.s. trend bad (7.07, *p*=0.06)	n.d.	n.s. trend bad (7.25, *p*=0.079)
SPP1	209875_s_at	n.s.	n.s.	**bad (7.33, *p*=0.042)**	**bad (6.94, *p*=0.046)**
TGFβ1	203084_at	**bad (10.3, *p*=0.034)**	n.s.	n.s.	n.s.
	203085_s_at	n.s. trend good (692.58, *p*=0.06)	**bad (9.95, *p*=0.046)**	n.s.	n.s.
TNFα	207113_s_at	n.s.	**bad (5.82, *p*=0.036)**	n.s. trend good (8.53, *p*=0.059)	n.s.
TNFRSF11A	207037_at	n.s. trend bad (4.93, *p*=0.097)	n.s.	**good (7.14, *p*=0.047)**	**bad (6.01, *p*=0.022)**
	238846_at	n.d.	n.s.	n.d.	**bad (5.67, *p*=0.031)**
TNFRSF11B	204932_at	n.s.	n.s.	n.s.	n.s.
	204933_s_at	n.s. trend bad (0.8, *p*=0.075)	n.s.	n.s. trend bad (6.57, *p*=0.077)	n.s.
